# SHORT-TERM DIESEL EXHAUST PARTICLE NEUROTOXICITY MEDIATED BY TLR4 ACROSS AGE AND SEX

**DOI:** 10.1093/geroni/igac059.3008

**Published:** 2022-12-20

**Authors:** Kristina Shkirkova, Hongqiao Zhang, Arnold Diaz, Wendy Mack, Constantinos Sioutas, Caleb Finch, Christian Pike, William Mack

**Affiliations:** USC Keck School of Medicine, Los Angeles, California, United States; USC, Los Angeles, California, United States; USC, Los Angeles, Armed Forces - AP, United States; USC, Los Angeles, California, United States; USC, Los Angeles, California, United States; USC, Los Angeles, California, United States; USC, Los Angeles, California, United States; USC, Los Angeles, California, United States

## Abstract

Experimental animal exposures show neurotoxic effects of diesel in young mice even after short-term exposures. We hypothesize that neuroinflammatory effects of diesel exhaust particles (DEP) are mediated by toll-like receptor 4 (TLR4) activation in brain microglia. We studied both sexes of young (2 months) and middle-aged (18 months) TLR4 flx/flx (CX3CR1CreER +/-) mice, in which macrophage-specific TLR4 deletion is induced by tamoxifen. DEP from National Institute of Science and Technology (NIST 2975) was suspended in pure water and re-aerosolized for 5 hr exposure at 100 µg/m3 concentration. Experimental groups were Filtered Air+Corn Oil, DEP+Corn Oil, Filtered Air+Tamoxifen, DEP+Tamoxifen. We studied markers of inflammation, oxidative stress, and microglia activation in the white matter of corpus callosum. At baseline, middle-aged mice showed higher levels of microglia activation (Iba-1), complement activation (C5, C5a), and oxidative stress (8-OHdG, 4HNE). DEP significantly increased microglial activation, inflammation, and oxidative stress. TLR4 knockdown showed a rescue effect in DEP group for Iba-1 and 8-OHdG in young but not in middle-aged mice. Both C5a and 4HNE were rescued by TLR4 knockdown in young and middle-aged mice, possibly with less robust effects in older mice. No significant sex effects were observed. Middle-aged mice have higher levels of baseline white matter inflammation and oxidative stress. DEP exposure caused robust neuroinflammatory and oxidative responses in white matter across ages. TLR4 knockdown attenuated DEP caused neuroinflammatory and oxidative responses, suggesting that microglia play an important role in DEP neurotoxicity.

